# Unusual presentation of an antrochoanal polyp: a case report

**DOI:** 10.11604/pamj.2017.26.233.12054

**Published:** 2017-04-25

**Authors:** Moncef Sellami, Abdelmonem Ghorbel

**Affiliations:** 1 Department of Otorhinolaryngology-Head and Neck Surgery, Habib Bourguiba University Hospital, Sfax, Tunisia; 2 Sfax Medical school, University of Sfax, Sfax, Tunisia

**Keywords:** Antrochoanal polyp, maxillary sinus, nasal Polyps

## Image in medicine

Antrochoanal polyps are benign lesions originating from the mucosa of the maxillary sinus and gradually prolapse through the medial wall of the maxillary sinus into the nasal cavity increasing in size to the choana and nasopharynx. Surgery is the usual treatment for antrochoanal polyps. We present the case of a 41-year-old woman who presented with a history of a progressive right nasal obstruction associated with a five-month history of sensation of a foreign body in the throat. Oral examination revealed a polypoid mass hanging from the nasopharynx into the oropharynx. Nasal endoscopy showed that the mass arose from the right middle meatus and extended into the nasal cavity and then into the nasopharynx. The computed tomography showed a complete opacification of the right maxillary sinus and a soft-tissue mass filling the low part of the right nasal cavity extended posteriorly through the right choana, filling up the nasopharynx and extending to the oropharynx. The polyp was removed under general anesthesia using a functional endoscopic sinus surgery and a large middle meatus antrostomy was performed. The patient made an uneventful post-operative recovery and was discharged home the following day. Histologic analysis confirmed that the mass was an antrochoanal polyp. The patient remained asymptomatic and disease-free at follow-up 12 months later.

**Figure 1:**
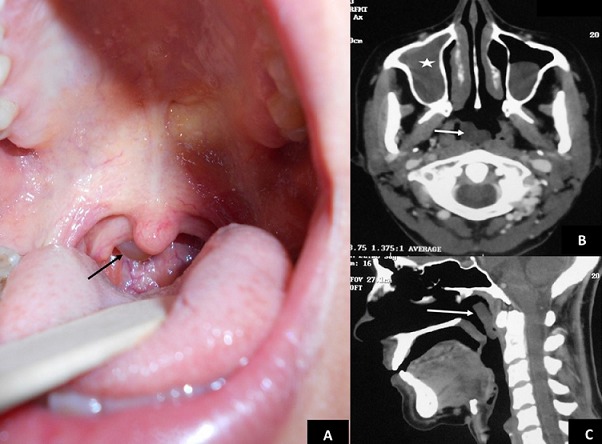
(A) clinical examination showing a pale, spherical mass hanging in the oropharynx (arrow); (B) axial computed tomography showed the complete opacification of the right maxillary sinus (star) and a polypoid mass of the nasopharynx (arrow); (C) sagittal computed tomography showed a polypoid mass of the nasopharynx (arrow)

